# Supplementary material for: Human health risk assessment of arsenic and potentially toxic elements exposure in bread and wheat flour in Northeast Iran
Source: PLoS One. 2025 Jul 23;20(7):e0327652. doi: 10.1371/journal.pone.0327652 (PMC12286368; doi:10.1371/journal.pone.0327652)
Supplement: S1 Table — (DOCX) [file pone.0327652.s002.docx]

Table S1. LOD, wavelength and recovery rate of ICP-OES for different elements

| Heavy metals | Pb | Hg | V | Co | Cd | As | Al | Zn | Ni | Cr | Cu | Fe |
| --- | --- | --- | --- | --- | --- | --- | --- | --- | --- | --- | --- | --- |
| LOD  (mg/kg) | 0.01 | 0.01 | 0.06 | 0.02 | 0.01 | 0.02 | 0.18 | 0.1 | 0.012 | 0.06 | 0.1 | 0.06 |
| Wavelength (nm) | 220.353 | 184.950 | 292.402 | 228.616 | 214.438 | 189.042 | 398.1 | 206.200 | 201.604 | 205.618 | 224.700 | 259.941 |
| Recovery (%) | 93 | 80.46 | 96 | 95.96 | 97.06 | 82.27 | 100.47 | 86.12 | 94.72 | 97.85 | 95.26 | 95.57 |
