# Supplementary material for: Human health risk assessment of arsenic and potentially toxic elements exposure in bread and wheat flour in Northeast Iran
Source: PLoS One. 2025 Jul 23;20(7):e0327652. doi: 10.1371/journal.pone.0327652 (PMC12286368; doi:10.1371/journal.pone.0327652)
Supplement: S2 Table — (DOCX) [file pone.0327652.s003.docx]

**Table S2. Mean concentrations of arsenic and heavy metals (mg kg^−1^) in flour, dough, and bread samples collected in this study.**

| **Site** | **Sample** | **Al**  **Mean±SD** | **As**  **Mean ±SD** | **Cd**  **Mean ±SD** | **Co**  **Mean ±SD** | **Cr**  **Mean ±SD** | **Cu**  **Mean ±SD** | **Fe**  **Mean±**  **SD** | **Hg**  **Mean ±SD** | **Ni**  **Mean ±SD** | **Pb**  **Mean ±SD** | **Zn**  **Mean ±SD** | **V**  **Mean ±SD** |
| --- | --- | --- | --- | --- | --- | --- | --- | --- | --- | --- | --- | --- | --- |
| **South** | **Flour** | **2.88±0.86** | **0.51±0.09** | **0.026±0.01** | **0.06±0.04** | **0.11±0.07** | **1.65±0.76** | **45.20±31.22** | **ND** | **0.09±0.05** | **ND** | **8.99±3.43** | **0.02±0.06** |
|  | **Dough** | **3.96±1.00** | **0.51±0.08** | **0.028±0.00** | **0.06±0.04** | **0.20±0.10** | **1.95±0.63** | **47.41±29.68** | **ND** | **0.13±0.06** | **ND** | **9.92±2.69** | **0.033±0.05** |
|  | **Bread** | **3.93±0.95** | **0.52±0.08** | **0.028±0.01** | **0.04±0.04** | **0.15±0.08** | **1.88±0.85** | **49.97±31.39** | **ND** | **0.16±0.08** | **ND** | **9.98±2.38** | **0.038±0.03** |
| **East** | **Flour** | **2.47±0.63** | **0.50±0.08** | **0.019±0.01** | **0.02±0.03** | **0.22±0.26** | **1.86±0.50** | **33.42±14.16** | **ND** | **0.12±0.04** | **ND** | **9.10±1.97** | **0.042±0.06** |
|  | **Dough** | **4.56±1.55** | **0.52±0.37** | **0.023±0.01** | **0.04±0.04** | **0.29±0.16** | **2.17±0.93** | **43.43±18.02** | **ND** | **0.16±0.17** | **ND** | **10.68±2.81** | **0.071±0.43** |
|  | **Bread** | **4.53±1.03** | **0.53±0.07** | **0.025±0.00** | **0.04±0.04** | **0.22±0.17** | **2.10±0.52** | **42.99±18.19** | **ND** | **0.17±0.07** | **ND** | **10.08±2.07** | **0.08±0.07** |
| **North** | **Flour** | **2.86±1.50** | **0.36±0.13** | **0.019±0.01** | **0.01±0.02** | **0.33±0.19** | **2.17±0.60** | **40.17±28.32** | **ND** | **0.08±0.09** | **ND** | **7.65±2.74** | **0.00±0.00** |
|  | **Dough** | **3.25±1.12** | **0.37±0.17** | **0.024±0.01** | **0.01±0.01** | **0.32±0.20** | **2.62±0.79** | **44.43±26.68** | **ND** | **0.12±0.13** | **ND** | **9.18±3.03** | **0.017±0.04** |
|  | **Bread** | **3.40±1.19** | **0.41±0.15** | **0.025±0.01** | **0.01±0.03** | **0.30±0.24** | **2.73±0.65** | **44.78±22.62** | **ND** | **0.13±0.12** | **ND** | **9.24±2.66** | **0.19±0.02** |
| **West** | **Flour** | **2.95±1.46** | **0.43±0.09** | **0.023±0.01** | **0.00±0.01** | **0.23±0.21** | **2.62±0.98** | **47.14±31.15** | **ND** | **0.042±0.13** | **ND** | **9.39±4.61** | **0.13±0.04** |
|  | **Dough** | **3.90±1.50** | **0.45±0.12** | **0.028±0.00** | **0.01±0.01** | **0.27±0.25** | **2.51±0.38** | **43.77±27.99** | **ND** | **0.055±0.12** | **ND** | **9.20±1.91** | **0.162±0.07** |
|  | **Bread** | **3.95±1.34** | **0.46±0.11** | **0.028±0.00** | **0.01±0.02** | **0.37±0.35** | **2.52±0.36** | **44.73±30.70** | **ND** | **0.06±0.16** | **ND** | **9.22±1.82** | **0.17±0.07** |
| **Center** | **Flour** | **2.76±0.71** | **0.49±0.04** | **0.033±0.00** | **0.00±0.00** | **0.41±0.25** | **2.43±0.57** | **50.45±29.70** | **ND** | **0.043±0.12** | **ND** | **8.84±2.71** | **0.071±0.04** |
|  | **Dough** | **3.96±1.00** | **0.53±0.06** | **0.033±0.00** | **0.01±0.02** | **0.39±0.22** | **2.54±0.56** | **56.40±39.56** | **ND** | **0.11±0.24** | **ND** | **9.72±2.64** | **0.082±0.07** |
|  | **Bread** | **3.92±1.13** | **0.53±0.10** | **0.031±0.00** | **0.01±0.02** | **0.47±0.26** | **2.53±0.54** | **58.94±29.25** | **ND** | **0.082±0.28** | **ND** | **9.71±2.42** | **0.11±0.07** |
| **Mean** | **Flour (n=90)** | **2.79±1.09** | **0.46±0.11** | **0.02±0.01** | **0.02±0.03** | **0.26±0.23** | **2.15±0.77** | **43.25±27.66** | **ND** | **0.13±0.09** | **ND** | **8.79±3.20** | **0.14±0.86** |
|  | **Dough**  **(n=90)** | **3.93±1.30** | **0.49±0.20** | **0.02±0.01** | **0.03±0.03** | **0.29±0.20** | **2.36±0.72** | **47.10±28.94** | **ND** | **0.11±0.16** | **ND** | **9.74±2.46** | **0.15±0.20** |
|  | **Bread (n=90)** | **3.94±1.16** | **0.49±0.11** | **0.02±0.01** | **0.029±0.03** | **0.30±0.26** | **2.36±0.65** | **47.68±26.93** | **ND** | **0.12±0.16** | **ND** | **9.76±2.26** | **0.15±0.06** |
| **^a^p-value** | **Flour** | **0.743** | **<0.001*** | **0.054** | **<0.001** | **0.001** | **<0.1** | **0.450** | **---** | **0.001** | **---** | **0.465** | **0.001** |
|  | **Dough** | **0.111** | **0.002** | **0.132** | **<0.001** | **0.044** | **0.4** | **0.715** | **---** | **<0.001** | **---** | **0.648** | **0.001** |
|  | **Bread** | **0.186** | **0.044** | **0.301** | **0.004** | **0.002** | **0.1** | **0.343** | **---** | **0.009** | **---** | **0.876** | **0.001** |

a: Statistically significant differences in the concentration of heavy metals in flour, dough, and bread samples from various areas were analyzed using the Kruskal-Wallis test.

* One-way ANOVA test

n: number of samples

ND: Not Detected
