# Supplementary material for: Human health risk assessment of arsenic and potentially toxic elements exposure in bread and wheat flour in Northeast Iran
Source: PLoS One. 2025 Jul 23;20(7):e0327652. doi: 10.1371/journal.pone.0327652 (PMC12286368; doi:10.1371/journal.pone.0327652)
Supplement: S3 Table — (DOCX) [file pone.0327652.s004.docx]

Table S3. Mean Concentration of Heavy Metals in Various Types of Bread Offered in Mashhad City

| **Type of bread** | **Al**  **(mg/kg)±SD** | **As**  **(mg/kg)±SD** | **Cd**  **(mg/kg)±SD** | **Co**  **(mg/kg)±SD** | **Cr**  **(mg/kg)±SD** | **Cu**  **(mg/kg)±SD** | **Fe**  **(mg/kg)±SD** | **Ni**  **(mg/kg)±SD** | **Zn**  **(mg/kg)±SD** | **V**  **(mg/kg)±SD** |
| --- | --- | --- | --- | --- | --- | --- | --- | --- | --- | --- |
| **Barbari (n=30)** | **36/3±08/1** | **46/0±11/0** | **02/0±01/0** | **02/0±03/0** | **26/0±25/0** | **17/2±75/0** | **00/44±56/31** | **11/0±15/0** | **84/8±82/2** | **04/0±06/0** |
| **Sangak (n=30)** | **74/3±22/1** | **51/0±19/0** | **02/0±01/0** | **03/0±03/0** | **27/0±18/0** | **38/2±81/0** | **75/43±65/22** | **09/0±14/0** | **46/10±71/2** | **16/0±88/0** |
| **lavash**  **(n=30)** | **42/3±49/1** | **46/0±12/0** | **02/0±01/0** | **02/0±03/0** | **33/0±24/0** | **20/2±56/0** | **15/50±40/28** | **11/0±13/0** | **75/8±38/2** | **06/0±07/0** |
| **^a^p-value** | **11/0** | **17/0** | **08/0** | **14/0** | **21/0** | **11/0** | **34/0** | **42/0** | **06/0** | **56/0** |

a: A statistically significant difference in the concentrations of heavy metals among the three types of bread was assessed using a One-Way ANOVA test.
