# Supplementary material for: Human health risk assessment of arsenic and potentially toxic elements exposure in bread and wheat flour in Northeast Iran
Source: PLoS One. 2025 Jul 23;20(7):e0327652. doi: 10.1371/journal.pone.0327652 (PMC12286368; doi:10.1371/journal.pone.0327652)
Supplement: S4 Table — (DOCX) [file pone.0327652.s005.docx]

Table S4. Permissible limits (mg/kg) for heavy metals in food.

|  | Standard | |
| --- | --- | --- |
| Heavy metal | **WHO/FAO*** | **Iranian National Standardization Organization (INSO)**  Standard No. 12968 |
| Cd | 0.003 | 0.03 |
| Pb | 0.3 | 0.05 |
| As | 0.15 | - |
| Hg | 0.005 | - |
| Fe | 20 | - |
| Al | 0.237 | - |
| V | - | - |
| Ni | 5 | - |
| Zn | 50 | - |
| Cr | 0.1 | - |
| Co | 0.1 | - |
| Cu | 10 | - |

*JECFA W. Summary and Conclusions of the 61st Meeting of the Joint FAO/WHO Expert Committee on Food Additives. JECFA WHO. 2003:1-188.

Codex Alimentarius Commission. (2016). General Standard for Contaminants and Toxins in Food and Feed. CODEX STAN 193–1995. Codex Alimentarius Commission.
