# Supplementary material for: Human health risk assessment of arsenic and potentially toxic elements exposure in bread and wheat flour in Northeast Iran
Source: PLoS One. 2025 Jul 23;20(7):e0327652. doi: 10.1371/journal.pone.0327652 (PMC12286368; doi:10.1371/journal.pone.0327652)
Supplement: S5 Table — (DOCX) [file pone.0327652.s006.docx]

Table S5. Average chronic daily intake of heavy metals through bread consumption for children and adults (mg per kilogram of body weight per day)

| **Metals** | **South** | | **East** | | **North** | | **West** | | **Center** | | **PTDI^^[[1]](#footnote-1)^^** | **MTDI^^[[2]](#footnote-2)^^** |
| --- | --- | --- | --- | --- | --- | --- | --- | --- | --- | --- | --- | --- |
|  | **Adult** | **Child** | **Adult** | **Child** | **Adult** | **Child** | **Adult** | **Child** | **Adult** | **Child** |  |  |
| **Al** | **0.0209** | **0.0366** | **0.0258** | **0.0451** | **0.0204** | **0.0358** | **0.0237** | **0.0415** | **0.0232** | **0.0407** | **0.143** | **-** |
| **As** | **0.0031** | **0.0039** | **0.0030** | **0.0038** | **0.0024** | **0.0030** | **0.0028** | **0.0035** | **0.0032** | **0.0040** | **0.0021** | **0.0018** |
| **Cd** | **0.0002** | **0.0002** | **0.0002** | **0.0002** | **0.0001** | **0.0002** | **0.0002** | **0.0002** | **0.0002** | **0.0002** | **0.00066** | **0.0008** |
| **Co** | **0.0003** | **0.0003** | **0.0003** | **0.0003** | **0.0001** | **0.0001** | **0.0001** | **0.0001** | **0.0001** | **0.0001** | **0.5** | **0.3** |
| **Cr** | **0.0009** | **0.0011** | **0.0014** | **0.0017** | **0.0018** | **0.0023** | **0.0022** | **0.0022** | **0.0028** | **0.0036** | **0.003** | **0.0028** |
| **Cu** | **0.0113** | **0.0142** | **0.0120** | **0.0150** | **0.0132** | **0.0165** | **0.0148** | **0.0185** | **0.0152** | **0.0192** | **0.5** | **0.1667** |
| **Fe** | **0.2999** | **0.3748** | **0.2520** | **0.3150** | **0.2567** | **0.3209** | **0.2684** | **0.3355** | **0.3537** | **0.4421** | **0.8** | **0.8** |
| **Ni** | **0.0006** | **0.0007** | **0.0009** | **0.0011** | **0.0006** | **0.0008** | **0.0004** | **0.0005** | **0.0012** | **0.0015** | **0.005** | **0.0043** |
| **Zn** | **0.0575** | **0.0719** | **0.0605** | **0.0757** | **0.0513** | **0.0641** | **0.0522** | **0.0691** | **0.0577** | **0.0721** | **1** | **0.3** |
| **V** | **0.0001** | **0.0001** | **0.0002** | **0.0003** | **0.0000** | **0.0000** | **0.0008** | **0.0010** | **0.0004** | **0.0005** | **-** | **-** |

Provisional tolerable daily intake value

^2^ Maximum tolerable daily intake

1. Provisional tolerable daily intake value [↑](#footnote-ref-1)
2. Maximum tolerable daily intake [↑](#footnote-ref-2)
