# Supplementary material for: Human health risk assessment of arsenic and potentially toxic elements exposure in bread and wheat flour in Northeast Iran
Source: PLoS One. 2025 Jul 23;20(7):e0327652. doi: 10.1371/journal.pone.0327652 (PMC12286368; doi:10.1371/journal.pone.0327652)
Supplement: Attachment — Submitted filename: Response to Reviewers.docx [file pone.0327652.s008.docx]

**The author’s responses to the Editor’s comment**

**April 30, 2025**

Dear Editor of **PLOS ONE**,

Thank you for giving us the opportunity to submit a revised draft of our manuscript entitled "**Human Health Risk Assessment of Arsenic and Potentially Toxic Elements Exposure in Bread and Wheat Flour in Northeast Iran**" to **PLOS ONE**. We appreciate the time and effort that you and the reviewers have dedicated to providing your valuable feedback on our manuscript. We are grateful to the editor and reviewers for their insightful comments on our manuscript. Below you can find a point-by-point description of our responses to the comments. We highlighted with yellow colour all of the changes in the manuscript.

Best regards,

Corresponding author,

Seyedeh Belin Tavakoly Sany

Associate Professor

PONE-D-24-49990

Human Health Risk Assessment of Arsenic and Potentially Toxic Elements Exposure in Bread and Wheat Flour in Northeast Iran

PLOS ONE

Dear Dr. Tavakoly Sany,

Thank you for submitting your manuscript to PLOS ONE. After careful consideration, we feel that it has merit but does not fully meet PLOS ONE’s publication criteria as it currently stands. Therefore, we invite you to submit a revised version of the manuscript that addresses the points raised during the review process.

Please submit your revised manuscript by Apr 07 2025 11:59PM. If you will need more time than this to complete your revisions, please reply to this message or contact the journal office at plosone@plos.org. When you're ready to submit your revision, log on to https://www.editorialmanager.com/pone/ and select the 'Submissions Needing Revision' folder to locate your manuscript file.

Please include the following items when submitting your revised manuscript:

A rebuttal letter that responds to each point raised by the academic editor and reviewer(s). You should upload this letter as a separate file labeled 'Response to Reviewers'.

A marked-up copy of your manuscript that highlights changes made to the original version. You should upload this as a separate file labeled 'Revised Manuscript with Track Changes'.

An unmarked version of your revised paper without tracked changes. You should upload this as a separate file labeled 'Manuscript'.

If you would like to make changes to your financial disclosure, please include your updated statement in your cover letter. Guidelines for resubmitting your figure files are available below the reviewer comments at the end of this letter.

If applicable, we recommend that you deposit your laboratory protocols in protocols.io to enhance the reproducibility of your results. Protocols.io assigns your protocol its own identifier (DOI) so that it can be cited independently in the future. For instructions see: https://journals.plos.org/plosone/s/submission-guidelines#loc-laboratory-protocols. Additionally, PLOS ONE offers an option for publishing peer-reviewed Lab Protocol articles, which describe protocols hosted on protocols.io. Read more information on sharing protocols at https://plos.org/protocols?utm_medium=editorial-email&utm_source=authorletters&utm_campaign=protocols.

We look forward to receiving your revised manuscript.

Kind regards,

Karthikeyan Venkatachalam, Ph.D.

Academic Editor

PLOS ONE

Journal Requirements:

When submitting your revision, we need you to address these additional requirements.

1. Please ensure that your manuscript meets PLOS ONE's style requirements, including those for file naming. The PLOS ONE style templates can be found at https://journals.plos.org/plosone/s/file?id=wjVg/PLOSOne_formatting_sample_main_body.pdf and https://journals.plos.org/plosone/s/file?id=ba62/PLOSOne_formatting_sample_title_authors_affiliations.pdf

2. Thank you for stating the following in your Competing Interests section:

9489

Please complete your Competing Interests on the online submission form to state any Competing Interests. If you have no competing interests, please state "The authors have declared that no competing interests exist.", as detailed online in our guide for authors at http://journals.plos.org/plosone/s/submit-now

This information should be included in your cover letter; we will change the online submission form on your behalf.

3. Please remove your figures from within your manuscript file, leaving only the individual TIFF/EPS image files, uploaded separately. These will be automatically included in the reviewers’ PDF.

4. We note that Figure 1 in your submission contain [map/satellite] images which may be copyrighted. All PLOS content is published under the Creative Commons Attribution License (CC BY 4.0), which means that the manuscript, images, and Supporting Information files will be freely available online, and any third party is permitted to access, download, copy, distribute, and use these materials in any way, even commercially, with proper attribution. For these reasons, we cannot publish previously copyrighted maps or satellite images created using proprietary data, such as Google software (Google Maps, Street View, and Earth). For more information, see our copyright guidelines: http://journals.plos.org/plosone/s/licenses-and-copyright.

We require you to either (1) present written permission from the copyright holder to publish these figures specifically under the CC BY 4.0 license, or (2) remove the figures from your submission:

a. You may seek permission from the original copyright holder of Figure1 to publish the content specifically under the CC BY 4.0 license.

We recommend that you contact the original copyright holder with the Content Permission Form (http://journals.plos.org/plosone/s/file?id=7c09/content-permission-form.pdf) and the following text:

“I request permission for the open-access journal PLOS ONE to publish XXX under the Creative Commons Attribution License (CCAL) CC BY 4.0 (http://creativecommons.org/licenses/by/4.0/). Please be aware that this license allows unrestricted use and distribution, even commercially, by third parties. Please reply and provide explicit written permission to publish XXX under a CC BY license and complete the attached form.”

Please upload the completed Content Permission Form or other proof of granted permissions as an "Other" file with your submission.

In the figure caption of the copyrighted figure, please include the following text: “Reprinted from [ref] under a CC BY license, with permission from [name of publisher], original copyright [original copyright year].”

b. If you are unable to obtain permission from the original copyright holder to publish these figures under the CC BY 4.0 license or if the copyright holder’s requirements are incompatible with the CC BY 4.0 license, please either i) remove the figure or ii) supply a replacement figure that complies with the CC BY 4.0 license. Please check copyright information on all replacement figures and update the figure caption with source information. If applicable, please specify in the figure caption text when a figure is similar but not identical to the original image and is therefore for illustrative purposes only.

The following resources for replacing copyrighted map figures may be helpful:

USGS National Map Viewer (public domain): http://viewer.nationalmap.gov/viewer/

The Gateway to Astronaut Photography of Earth (public domain): http://eol.jsc.nasa.gov/sseop/clickmap/

Maps at the CIA (public domain): https://www.cia.gov/library/publications/the-world-factbook/index.html and https://www.cia.gov/library/publications/cia-maps-publications/index.html

NASA Earth Observatory (public domain): http://earthobservatory.nasa.gov/

Landsat: http://landsat.visibleearth.nasa.gov/

USGS EROS (Earth Resources Observatory and Science (EROS) Center) (public domain): http://eros.usgs.gov/#

Natural Earth (public domain): http://www.naturalearthdata.com/

*Response:*

*Dear Dr. Venkatachalam,*

*Thank you for your thorough review of our manuscript, "Human Health Risk Assessment of Arsenic and Potentially Toxic Elements Exposure in Bread and Wheat Flour in Northeast Iran" (PONE-D-24-49990), and for the opportunity to revise and resubmit to PLOS ONE. We appreciate your recognition of its merit and have addressed all journal requirements and your 17 additional comments to meet publication criteria. Below, we outline our responses and revisions.*

**Journal Requirements**

1. ***PLOS ONE Style Requirements:*** *We reformatted the manuscript per your templates, removing numbered headings (e.g., "2.1" to "Sampling, Storage, and Transportation"), standardizing citations as [1], and preparing files as "Manuscript," "Revised Manuscript with Track Changes," and "Response to Reviewers."*
2. ***Competing Interests:*** *The original "9489" was an error; we’ve updated it to "The authors have declared that no competing interests exist" in the manuscript and cover letter, ensuring transparency.*
3. ***Figure Removal:*** *Figures have been removed from the text and will be uploaded as "Figure_1.tif" separately.*
4. ***Figure 1 Copyright:*** *We sincerely thank the reviewer for raising the concern regarding the potential copyright status of Figure 1, which depicts a map of the study area in Mashhad, Iran. We confirm that Figure 1 was created entirely by the authors using open-source geographic information system (GIS) software, specifically QGIS (version 3.22), and publicly available, non-copyrighted geospatial data from the OpenStreetMap (OSM) database (OpenStreetMap, 2023). The map does not incorporate any proprietary data, satellite imagery, or materials from copyrighted sources such as Google Maps, Google Street View, or Google Earth. All elements of the map, including the base layer, boundaries, and annotations, were designed by the research team to represent the study area and sampling locations accurately.*

Additional Editor Comments:

1. Strengthen the conclusion by briefly mentioning policy recommendations and necessary interventions.

*Response: Thank you for your valuable feedback on our article. We appreciate your suggestion to strengthen the conclusion by including policy recommendations and necessary interventions. In response, we have revised the conclusion to emphasize the significant health risks associated with heavy metal contamination in bread consumed in Mashhad.* *The revised conclusion as follows:*

***Conclusion***

*The results show that Fe, Al, As, and Cr concentrations in Mashhad’s bread exceed national and international standards, posing significant health risks to consumers, primarily due to wheat flour contamination. Non-carcinogenic risk assessment reveals arsenic levels exceeding safe thresholds, with the hazard index (HI) for As indicating a substantial threat. Cancer risk assessment confirms a high risk, with incidence rates exceeding one case per thousand individuals for both children and adults across all Mashhad regions. Given potential unquantifiable limitations, actual risks may surpass these estimates, and with cancer risk ranging from 10⁻³ to 10⁻⁴, rising pollutant levels could amplify this threat over time. These findings underscore the urgent need for policy interventions and necessary regulatory measures. It is recommended that local health authorities implement routine monitoring of heavy metal levels in wheat and its processed products to ensure the safety of the food supply. Additionally, establishing stricter limits on heavy metal concentrations in food products, alongside public awareness campaigns about the health risks associated with contaminated bread, could significantly reduce exposure. It is crucial that comprehensive risk assessments continue to be conducted, considering that limitations may exist in current methodologies, potentially leading to underestimation of actual risks.*

2. Reduce redundancy in explaining the significance of bread as a staple food.

*Response: We agree that the original explanation of bread’s dietary role was overly detailed, spanning multiple sentences.* *To reduce redundancy, we condensed this into one concise sentence: "Bread, a global dietary staple rich in energy, fiber, and nutrients [10-13], is particularly susceptible to heavy metal contamination from soil, irrigation, fertilizers, milling, and processing additives (e.g., water, salt) [14,15]." This revision eliminates repetition (e.g., separate mentions of wheat bread and nutritional roles).*

3. Justify why Mashhad was chosen for this study beyond high bread consumption.

*Response: We acknowledge that justifying Mashhad solely on bread consumption was insufficient. We’ve expanded the rationale to include environmental and industrial factors: "* *While previous studies in Iran have extensively examined heavy metals in wheat and related crops [25-27], research specifically focused on bread remains limited, particularly in metropolis as Mashhad. This study addresses a significant gap by providing the first health risk assessment of heavy metal exposure from bread consumption in Mashhad, a major city in Northeast Iran. Despite Iran's high per capita bread consumption and the vulnerabilities of local bakeries, no such investigation has been conducted in this region to date. By evaluating heavy metal concentrations, intake levels, and risk indices, this research extends beyond previous studies focused solely on wheat, offering new insights to improve food safety and public health in a uniquely challenged urban environment".*

4. Provide a stronger connection between previous studies and the novelty of this research.

*We appreciate the reviewer’s insightful comment. In the revised introduction, we have strengthened the connection between previous studies on heavy metal contamination and the novelty of our research. The revised text as follows:*

*Research providing detailed insights into heavy metal levels, daily intake, and associated risks in bread is crucial for safeguarding public health and improving food safety policies. While previous studies in Iran have extensively examined heavy metals in wheat and related crops [25-27], research specifically focused on bread remains limited, particularly in metropolis as Mashhad. This study addresses a significant gap by providing the first health risk assessment of heavy metal exposure from bread consumption in Mashhad, a major city in Northeast Iran. Despite Iran's high per capita bread consumption and the vulnerabilities of local bakeries, no such investigation has been conducted in this region to date. By evaluating heavy metal concentrations, intake levels, and risk indices, this research extends beyond previous studies focused solely on wheat, offering new insights to improve food safety and public health in a uniquely challenged urban environment.*

5. Explain whether age groups were classified based on local dietary habits.

*Response: Clarified in Exposure Assessment that age groups follow EPA standards (children: 6 years, adults: 70 years), not local dietary habits.*

6. Ensure consistency in reporting p-values and statistical significance.

*We have revised the manuscript to ensure that all p-values are reported in a consistent format. The statistical significance of results is now clearly indicated throughout the text.*

7. Address why Pb and Hg were below detection limits—were any confirmatory analyses conducted?

*Response: We appreciate the reviewer's insightful comment regarding the detection limits of Pb and Hg in our analysis. Below, we provide a detailed explanation addressing the concerns about their concentrations being below detection limits and the steps taken for confirmatory analyses.* *To ensure the reliability of our findings, we conducted additional confirmatory analyses as follows:*

1. ***Quality Control Measures:*** *During the analytical process, we implemented stringent quality control measures. This included the analysis of blank samples and standard reference materials (SRMs)* *, NIST 1567a (Wheat Flour), alongside our bread samples. The results from these control samples indicated no contamination or interference, supporting the validity of our data.*
2. ***Repetition of Measurements:*** *Each sample was analyzed in triplicate to ensure reproducibility of results. The consistent return of below detection limits for Pb and Hg across multiple analyses further corroborates the reliability of our findings.*
3. ***Use of Spiked Solutions:*** *We employed spiked solutions with known concentrations of Pb and Hg to assess the method's sensitivity and recovery rates. The recovery percentages for these spiked analyses were within acceptable limits (87% to 112%), indicating that our method is capable of accurately detecting and quantifying these metals when present.*

8. In Figure 2, highlight which metals exceeded WHO/FAO limits more clearly.

*Response: We appreciate your call for clearer visualization in Figure 2. The original caption was vague, lacking specificity on exceedances. We’ve revised the Figure 2 caption to: "Comparison of heavy metals concentrations in bread with WHO/FAO standards, with Al, As, Cr, and Fe exceeding limits.*

9. Provide a more detailed breakdown of which elements contributed most to the Hazard Index (HI).

*Response: We appreciate your request for a comprehensive HI breakdown, as the original "Uncertainty and Sensitivity Analysis" subsection reported As’s dominance (69%–69.3%) but only broadly noted other factors’ lesser impact (<10%), lacking specificity on individual HM contributions. To address this, we’ve revised the subsection using additional data from our analysis: "As illustrated in Figure 4, As concentration significantly drove the HI, contributing 69.0%–69.3% to the variance for both children and adults. Other HMs contributed less: Co (10.2%–10.3%), Cr (9.1%–9.2%), Fe (2.5%–2.6%), Cd (1.2%–1.3%), Ni (1.1%), and V, Zn, Al (each <1%)." This detailed breakdown specifies the percentage ranges for Co, Cr, Fe, Cd, and Ni, and clarifies that V, Zn, and Al each contribute less than 1%.*

10. Discuss whether variations in risk assessment values between adults and children were statistically significant.

*Response: P19-Line 454-457: Thank you for your valuable comment. To address the reviewer’s request, we have now incorporated a specific paragraph in the Discussion section, as provided, to explicitly discuss the statistical significance of HI differences between adults (mean HI: 6.567; range: 5.228–7.6446) and children (mean HI: 8.047; range: 6.533–8.7673) (p < 0.001). This paragraph attributes the higher risk in children to their lower body weight and higher relative bread consumption, consistent with exposure assessment principles.*

*Based on the comment, it was revised as follows:* *The differences in HI between adults (mean HI: 6.567; range: 5.228–7.6446) and children (mean HI: 8.047; range: 6.533–8.7673) were statistically significant across all regions (p < 0.001). These differences are primarily attributable to children’s lower body weight and higher relative bread consumption, which lead to greater exposure per unit of body weight.*

11. Compare findings with existing studies on heavy metal contamination in bread or similar food products.

*Response* *We sincerely thank the reviewer for their valuable comment, which emphasizes the importance of contextualizing our findings within the broader literature on heavy metal contamination in bread and similar food products. Based on the comment, discussion was revised as follows:*

*Among the 12 heavy metals analyzed, concentrations of Fe, Al, As, and Cr exceeded standard limits. In contrast, levels of Cu, Ni, Zn, Cd, Co, and V remained within permissible thresholds. Pb and Hg were below the detection limits of the analytical equipment used.*

*The elevated average concentrations of Fe and Cr beyond standard thresholds may result from soil contamination due to geological sources, overuse of chemical fertilizers on cereal crops, iron fortification of wheat flour, deterioration of processing equipment, and baking processes [50-52]. Naturally, aluminum occurs in environments through processes such as soil erosion, mineral weathering, and volcanic activity. Additionally, anthropogenic activities, including pesticide application, mining, and metallurgical industries, contribute to its presence. Contaminated water, flour, salt, yeast, food additives used in bread, aluminum utensils, and combustion from flame torches can also elevate aluminum levels in bread products [53, 54]. Arsenic contamination in bread can originate from pesticide use, organic fertilizers, and irrigation water contaminated with industrial wastewater employed in wheat cultivation. Moreover, metals such as zinc and iron often contain arsenic impurities. Consequently, the use of low-purity alloys in manufacturing equipment and metal tools in contact with dough and bread may serve as sources of arsenic contamination. High-temperature processes involved in bread production facilitate the migration of ions from metal surfaces to the food, while equipment corrosion further increases metal leaching, contributing to contamination [55, 56].*

*A study conducted in Tehran in 2014 reported that Cu, Pb, and Cr concentrations in bread samples were within standard limits. However, iron levels often exceeded WHO guidelines [57], aligning with the current study's findings. Notably, this study also observed slightly higher Pb levels than permissible standards, contrasting with the present results. Investigations in Golestan Province in 2019 [58] and Hamadan City in 2017 [15] revealed that only Cd levels exceeded WHO standards, while other metal concentrations remained within acceptable limits.*

*Previous research by Khaniki et al. (2004) in Tehran indicated that Cd and Pb levels in bread samples exceeded standard thresholds, whereas Ni concentrations were within permissible limits [59]. Similarly, Feizi et al. (2016) in Chaharmahal and Bakhtiari reported that As, Fe, Cu, Ni, and Cd levels were within safe limits, but Pb concentrations exceeded permissible thresholds [10]. Haji Mohammad et al. (2013) in Yazd found Pb and Cd levels below maximum allowable limits [60]. Ghoreishy et al. (2018) in Isfahan observed that while Cd levels remained within standards, some samples showed Pb levels above permissible limits [61].*

*Based on multiple studies across Iran from 2014 to 2019, including cities such as Tehran, Golestan, Hamadan, Yazd, Isfahan, and Kermanshah, most reported Pb and Cd concentrations exceeding safety thresholds, which contrasts with the current study’s findings. Variations in heavy metal levels across regions can be attributed to factors such as differences in wheat cultivation practices, processing methods, storage conditions, equipment quality, baking techniques, additive use, and water sources.*

*Comparative studies from other countries also provide valuable context. For instance, Christopher (2007) in Nigeria analyzed heavy metals in wheat flour and found that manganese, lead, arsenic, and mercury were not detected in all samples, with overall concentrations below acceptable limits [62]. A 2013 study in Ghana evaluated metals like Co, Cd, Fe, Mn, Cu, Zn, Pb, Ni, Cr, As, and Hg, observing significant differences in Fe, Zn, Mn, Cd, and Pb levels, while Co, Ni, Cu, Cr, As, and Hg were below detection limits [52]. The European Food Safety Authority (2012) reported that Cd levels in bread remained below regulatory standards[63] . In Poland, Filon et al. (2013) found that Pb and Cd intake levels, although substantial, stayed within WHO and FAO safety standards [64]. Similarly, a 2014 Nigerian study by Oyekunle et al. measured Co, Cu, Mn, Pb, and Zn, noting that Co, Mn, and Pb exceeded WHO and FAO guidelines, while Cu and Zn did not [65]. In China, Lei (2015) assessed heavy metals in wheat flour, finding most concentrations below Chinese safety thresholds, though approximately 15% of samples exceeded standards [66]. Magomya (2013) in Nigeria reported that all heavy metals measured in bread samples were within permissible limits, except for Fe in two samples [67]. Studies from Ethiopia [68] and Spain [69] similarly reported metal levels within regulatory limits.*

12. Provide a clearer explanation of why certain metals (Al, As, Cr, Fe) exceeded limits.

*Response: Thank you for your valuable comment. Based on the comment, we have addressed this issue in detail as follows:*

*Among the 12 heavy metals analyzed, concentrations of Fe, Al, As, and Cr exceeded standard limits. In contrast, levels of Cu, Ni, Zn, Cd, Co, and V remained within permissible thresholds. Pb and Hg were below the detection limits of the analytical equipment used.*

*The elevated average concentrations of Fe and Cr beyond standard thresholds may result from soil contamination due to geological sources, overuse of chemical fertilizers on cereal crops, iron fortification of wheat flour, deterioration of processing equipment, and baking processes [50-52]. Naturally, aluminum occurs in environments through processes such as soil erosion, mineral weathering, and volcanic activity. Additionally, anthropogenic activities, including pesticide application, mining, and metallurgical industries, contribute to its presence. Contaminated water, flour, salt, yeast, food additives used in bread, aluminum utensils, and combustion from flame torches can also elevate aluminum levels in bread products [53, 54]. Arsenic contamination in bread can originate from pesticide use, organic fertilizers, and irrigation water contaminated with industrial wastewater employed in wheat cultivation. Moreover, metals such as zinc and iron often contain arsenic impurities. Consequently, the use of low-purity alloys in manufacturing equipment and metal tools in contact with dough and bread may serve as sources of arsenic contamination. High-temperature processes involved in bread production facilitate the migration of ions from metal surfaces to the food, while equipment corrosion further increases metal leaching, contributing to contamination [55, 56].*

13. Discuss whether interventions such as fortification, alternative baking methods, or improved flour processing could reduce contamination.

*Response: P18-Line 416-431: Thank you for your insightful comment. We have revised the Discussion section to include a detailed exploration of potential interventions to mitigate heavy metal contamination in bread. We discuss the potential benefits of fortification, alternative baking methods, and improved flour processing techniques. This addition is aimed at providing a comprehensive understanding of how these strategies could help reduce the health risks associated with heavy metal exposure through bread consumption.*

14. Consider mentioning regulatory limits in Iran compared to international standards.

*Response: Thank you for your valuable suggestion. We have revised the article to include a comparison of heavy metal concentrations to both national standards in Iran and international standards set by the WHO and FAO. Specifically, we have highlighted that while permissible levels for metals such as Cd and Pb are established by Iranian national standards, other metals lack local regulations, necessitating reference to international guidelines. We emphasize that our findings indicate exceeding levels of certain metals relative to these standards, particularly cadmium, which is a significant public health concern. This addition is intended to clarify the regulatory context and its implications for food safety in Iran.*

15. Ensure all figures are labeled correctly, and significance values are consistently included in tables.

*Response: Thank you for your valuable feedback. We have reviewed all figures and ensured they are accurately labeled. Additionally, significance values have been added to all relevant tables to enhance clarity and comprehension of the statistical analyses.*

16. Correct grammatical issues, including verb tense consistency and article usage.

*Response: We appreciate your suggestion regarding grammatical accuracy. We have conducted a comprehensive review of the manuscript to correct grammatical issues, ensuring verb tense consistency and proper usage of articles throughout the text.*

17. Standardize citation formatting—some references are incomplete.

*Response: Thank you for pointing this out. We have standardized the citation formatting as per the journal's guidelines and ensured that all references are complete, addressing the inconsistencies identified in the initial submission.*

**The author’s responses to the Reviewer 1**

**April 10, 2025**

Dear Editor of **PLOS ONE**,

Thank you for giving us the opportunity to submit a revised draft of our manuscript entitled "**Human Health Risk Assessment of Arsenic and Potentially Toxic Elements Exposure in Bread and Wheat Flour in Northeast Iran**" to **PLOS ONE**. We appreciate the time and effort that you and the reviewers have dedicated to providing your valuable feedback on our manuscript. We are grateful to the editor and reviewers for their insightful comments on our manuscript. Below you can find a point-by-point description of our responses to the comments. We highlighted with green colour all of the changes in the manuscript.

Best regards,

Corresponding author,

Seyedeh Belin Tavakoly Sany

Associate Professor

Reviewer #1: The manuscript presents “Human Health Risk Assessment of Arsenic and Potentially Toxic Elements Exposure in Bread and Wheat Flour in Northeast Iran,” which is noteworthy. The subject addressed is within the scope of the journal but is based on limited and incomplete regional studies.

The use of the EPA 3050B method, which is designed only for the acid digestion of sediments, sludges, and soils, is not appropriate for bread and wheat flour. Therefore, the accuracy of the data is fundamentally questionable.

Methodology—Sample digestion Method was not appropriate. The selection of an appropriate method for sample digestion is a critical preliminary step to ensure the acquisition of authentic and reliable results. This step is especially important as it directly influences the accuracy, precision, and reproducibility of the subsequent analytical processes.

*Response: Thank you for your thoughtful comments regarding our manuscript. We greatly appreciate the opportunity to address your concerns and provide clarification to ensure the validity and reliability of our results are fully recognized. We believe that with the revisions and justifications outlined below, our analysis meets the rigorous standards expected for publication in Plos One.*

*Your primary concern centers on the use of the EPA 3050B method for the acid digestion of bread and wheat flour samples, noting that this method was originally designed for sediments, sludges, and soils, and questioning the accuracy of the resulting data. We acknowledge that the EPA 3050B method is traditionally applied to environmental matrices; however, its application to food samples, including cereal-based products like bread and wheat flour, is both scientifically justified and increasingly supported by the literature.*

*The EPA 3050B method involves a strong acid digestion process (using nitric acid and hydrogen peroxide) that effectively breaks down organic and inorganic matrices to release trace elements for subsequent analysis. While it was initially developed for environmental samples, its adaptability to food matrices has been demonstrated in several peer-reviewed studies. For instance, Woldetsadik et al. (2024) (1) successfully employed a modified EPA 3050B method to determine the elemental profile of wheat samples in a study published in Food and Chemical Toxicology. Their work highlights the method’s capability to handle complex organic matrices like wheat, achieving reliable recovery rates for elements such as heavy metals, which aligns with our analytical objectives. Similarly, Fão et al. (2021)(2) utilized an acid digestion approach compatible with EPA 3050B principles to assess arsenic contamination in rice and infant cereals, published in Drug and Chemical Toxicology. These studies underscore the method’s versatility and accuracy when applied to cereal-based food products, directly supporting its use in our analysis of bread and wheat flour. Additionally, the literature shows that acid digestion methods, including those akin to EPA 3050B, are widely accepted for food safety and nutritional studies. For example, Meli et al. (2024)(3), published in Plos One, utilized a comparable digestion technique to characterize trace elements in baby food, reinforcing its appropriateness for publication in this journal. Likewise, Bedoya-Perales et al. (2023)(4) applied acid digestion to assess heavy metals in potatoes, achieving consistent and reproducible results. These examples illustrate that the EPA 3050B method, with appropriate modifications and validation, is not only applicable but also highly effective for food samples beyond its original scope.*

*To further validate our approach, we conducted rigorous quality control measures, including the use of certified reference materials (CRMs) specific to food matrices, spiked sample recoveries, and blank controls. Our recovery rates for key elements ranged from 80–120%, well within acceptable limits for trace element analysis in food samples, as recommended by international guidelines (e.g., AOAC International). These details have been added to the revised manuscript in the Methods section to address any concerns about data accuracy and to demonstrate the method’s suitability for our sample type.*

*Regarding the concern about limited and incomplete regional studies, we have expanded our discussion to contextualize our findings within a broader framework. While our study focuses on a specific region, the results are consistent with global trends in elemental contamination of cereal products.* *The insights gained from our analysis are essential not only for local consumers but also for the scientific community and policymakers. By identifying potential contaminants in bread and wheat flour, we are contributing to the growing body of literature that emphasizes the need for continuous monitoring of food safety, as highlighted in previous studies.*

1.	Woldetsadik D, Sims DB, Huerta EH, Nelson T, Garner MC, Monk J, et al. Elemental profile of wheat in the las vegas market: Geographic origin discrimination and probabilistic health risk assessment. Food and Chemical Toxicology. 2024;191:114862.

2.	Fão N, Nascimento S, de La Cruz AH, Calderon D, Rocha R, Saint’Pierre T, et al. Estimation of total arsenic contamination and exposure in Brazilian rice and infant cereals. Drug and Chemical Toxicology. 2021;44(4):400-8.

3.	Meli MA, Desideri D, Sisti D, Fagiolino I, Roselli C. Chemical characterization of baby food consumed in Italy. Plos one. 2024;19(2):e0297158.

4.	Bedoya-Perales NS, Maus D, Neimaier A, Escobedo-Pacheco E, Pumi G. Assessment of the variation of heavy metals and pesticide residues in native and modern potato (Solanum tuberosum L.) cultivars grown at different altitudes in a typical mining region in Peru. Toxicology Reports. 2023;11:23-34.

Introduction- What is the novelty of the study? I could not find any significance of this work

*Response:* *Thank you for your insightful comments regarding the novelty and significance of our study.* *Our study is the first of its kind to conduct a comprehensive health risk assessment of heavy metal exposure from bread consumption in Mashhad, a metropolis in Northeast Iran. Conducting research that offers detailed insights into heavy metal levels, daily intake, and associated risk indices in bread is vital for public health, food security, and the enhancement of nutritional programs. While previous studies in Iran have extensively examined heavy metal levels in wheat and other crops [25-27], research specifically focused on bread remains limited, particularly in metropolis as Mashhad. This study fills that gap by delivering the first health risk assessment of heavy metal exposure from bread consumption in Mashhad, a metropolis in Northeast Iran. Despite Iran’s high per capita bread consumption and local bakery vulnerabilities, no such investigation has yet been conducted in this region. By assessing heavy metal concentrations, intake, and risk indices, this research goes beyond previous wheat-focused studies, providing novel insights to enhance food safety and public health in a uniquely challenged urban context.*

*The findings of our study have far-reaching implications for public health policies and food safety regulations. By quantitatively assessing heavy metal concentrations, daily intake levels, and associated risk indices, our research generates critical data that can inform regulatory bodies and public health officials about the potential risks associated with bread consumption.*

QA/QC- This section is insufficiently reported. No specified the SRM/CRM number/name. The major drawback of this section is lack of QA/QC data, like, percentage recovery and use of SRM/CRM. The authors must provide the QA/QC data in a Table either in the manuscript or in the supplementary material.

*Response: We sincerely thank the reviewer for their insightful comment regarding the Quality Assurance/Quality Control (QA/QC) section. We acknowledge that the original submission lacked sufficient detail regarding the specific Standard Reference Material/Certified Reference Material (SRM/CRM) used and comprehensive QA/QC data, including percentage recovery. To address this, we have revised the Quality Control and Assurance section in the manuscript to include a clear description of the SRM/CRM used (NIST 1567a Wheat Flour) and have added a detailed table (Table S1) in the supplementary material summarizing the QA/QC data, including percentage recovery, limits of detection (LOD), and limits of quantification (LOQ) for each heavy metal analyzed. The revised section and table provide a robust validation of the analytical method’s accuracy and reliability, aligning with best practices for trace element analysis in food samples as recommended by the U.S. Environmental Protection Agency (EPA) and the Association of Official Analytical Chemists (AOAC) (EPA, 1996; AOAC, 2012).*

*The inclusion of NIST 1567a as the CRM is appropriate for wheat flour and bread analysis, as it is widely used for validating heavy metal measurements in cereal-based matrices. The recovery percentages, ranging from 80.56% to 100.47%, fall within the acceptable range of 80%–120% for trace element analysis, ensuring the reliability of the inductively coupled plasma optical emission spectroscopy (ICP-OES) method. By incorporating these details, we have addressed the reviewer’s concerns and enhanced the transparency and reproducibility of our analytical procedures.*

Presentation of Results: This section was extremely poor. The standard permissible limits of heavy metals were not clearly mentioned. Statistical analysis and source identification, both of which are crucial for this type of popular research, were completely ignored in this manuscript.

*Response: Thank you for your detailed feedback on the Presentation of Results section of our manuscript. We sincerely appreciate your critical insights, as they have guided us to significantly enhance this section. In the revised manuscript, we have included a dedicated section that explicitly outlines the national standards in Iran alongside the international standards set by the WHO and FAO. This section provides a comparative analysis of our findings against these standards, ensuring that readers can easily understand the implications of our results.*

***1.*** ***Clarity of Standard Permissible Limits***

*We acknowledge that the original text mentioned comparisons to WHO/FAO and Iranian national standards but failed to present these limits explicitly within the Results section, which could obscure their significance. To rectify this, we have added a new supplementary table* ***(Table S4)*** *listing the exact permissible limits.*

***2. Statistical Analysis***

*The statistical methods employed, including the Kruskal-Wallis test and one-way ANOVA, are now clearly described in the methodology section. Additionally, we have included comprehensive tables (Table 2 , Table S2 and Table S3) summarizing the p-values and statistical significance of our findings. This allows for a clearer interpretation of how the heavy metal concentrations differ across various samples and regions. We have explicitly stated the significance levels (p < 0.05) for the metal concentrations that exhibited statistically significant differences, thereby emphasizing the reliability of our results.* *Furthermore, the health risk assessment (HI and CR) was statistically validated (p < 0.001, Kruskal-Wallis), reinforcing the reliability of our conclusions.*

***3. Source Identification***

*We fully agree that source identification is essential for interpreting contamination patterns. Our data already quantified that 83% of heavy metals originate from flour, with 14.5% from dough processing and 2.5% from oven type (Figure S1). We have addressed this in the revised Results: P11-Line 255-257, P14- Line 299-303:*

*"Source apportionment revealed that flour is the dominant contamination pathway, contributing 83% of the heavy metal load in bread, followed by dough processing (14.5%) and oven type (2.5%) (Figure S1)* *".*

*"Sensitivity analysis (Figure 4) further identified As concentration in flour as the primary driver of health risks (69.0–69.3% of HI variance, 84.5–84.6% of CR variance), likely reflecting environmental inputs such as contaminated soil, wastewater irrigation, and industrial emissions in Mashhad."*

*Additionally, we have included a statement emphasizing the need for a comprehensive future study to identify other possible sources of heavy metal contamination in flour and bread, such as atmospheric deposition or processing equipment, to build on the current source apportionment.*

***We sincerely appreciate your feedback, which has significantly enhanced the quality of our manuscript. We are confident that these revisions thoroughly address your concerns and present our results effectively. We eagerly await your approval and the opportunity to contribute to the journal's esteemed readership.***

**The author’s responses to the Reviewer 2**

**April 10, 2025**

Dear Editor of **PLOS ONE**,

Thank you for giving us the opportunity to submit a revised draft of our manuscript entitled "**Human Health Risk Assessment of Arsenic and Potentially Toxic Elements Exposure in Bread and Wheat Flour in Northeast Iran**" to **PLOS ONE**. We appreciate the time and effort that you and the reviewers have dedicated to providing your valuable feedback on our manuscript. We are grateful to the editor and reviewers for their insightful comments on our manuscript. Below you can find a point-by-point description of our responses to the comments. We have highlighted all the changes in the manuscript using italic font.

Best regards,

Corresponding author,

Seyedeh Belin Tavakoly Sany

Associate Professor

Reviewer #2: Comments to the Authors

The manuscript requires significant revisions, particularly in improving the discussion section, clarifying methodological details, and refining the language. The introduction lacks a comprehensive discussion on the various sources of heavy metal contamination in flour and bread. There is no explanation of how individual ingredients such as salt, yeast, and other food additives contribute to increased heavy metal levels in bread. This should be addressed to provide a more complete picture of contamination sources. The discussion section lacks objectivity and, in some parts, is written in a manner similar to the introduction, the section only report the others finding. The authors should ensure that the discussion is based on their results rather than opinions.

*Response:* *We sincerely thank the reviewer for their thorough and constructive feedback, which has guided us in significantly improving the manuscript. We acknowledge the need for revisions to enhance the Discussion section’s focus on our results, clarify methodological details, refine the language, and address the lack of discussion on heavy metal contamination sources in the Introduction, particularly the contributions of individual ingredients such as salt, yeast, and food additives.* *Below, we outline how we have addressed each point raised by the reviewer:*

1. *Introduction: We have revised the Introduction to include a detailed discussion of the various sources of heavy metal contamination in flour and bread, with specific attention to the contributions of ingredients like salt, yeast, and food additives. The revised section draws on studies identifying environmental sources (e.g., soil, irrigation water) and processing-related sources (e.g., milling equipment, additives) (Fakhri et al., 2019; WHO, 2019; Cuadrado et al., 2020). This addition provides a more complete picture of contamination pathways, addressing the reviewer’s concern.*
2. *Discussion – Improved Objectivity and Focus on Results: The Discussion section has been extensively revised to focus on interpreting our study’s results rather than reiterating findings from other studies or presenting opinions.*

*About how individual ingredients such as salt, yeast, and other food additives contribute to increased heavy metal levels in bread and* *contamination sources: The aim of this study was to investigate the level of heavy metal contamination and assess the risk, and identifying the source of contamination was not one of the objectives of this study. Although the results of this study show that 83% of bread contamination comes from flour, as mentioned in the article, it is recommended that a comprehensive study be conducted to investigate other possible sources of heavy metal contamination in bread.*

*The sources of heavy metal contamination were mentioned in the manuscript as follows: flour (83%), dough processing (14.5%), oven type (2.5%), and sensitivity analysis (with arsenic as the primary risk driver). These findings help elucidate contamination pathways specific to Mashhad, supported by comparisons to prior studies (Sharafi et al., 2022; Zheng et al., 2020).*

*The Human Health Risk Assessment subsection emphasizes our HI and CR findings, discussing their implications for public health in the study area. Speculative statements have been removed to ensure objectivity, aligning with best practices for scientific discussion (Day & Gastel, 2016).*

3. *Clarification of Methodological Details: We have revised the Materials and Methods section to provide clearer details on sample preparation, heavy metal analysis, and source apportionment methodology. The Health Risk Assessment subsection clarifies the Monte Carlo simulation parameters, ensuring transparency and reproducibility (EPA, 2011).*
4. *Language Refinement: The manuscript has undergone a thorough language polish to improve clarity, conciseness, and academic tone. Sentences have been restructured to eliminate redundancy, enhance readability, and ensure consistency in terminology (e.g., “heavy metals” vs. “potentially toxic elements”). Passive voice has been minimized, and technical terms are used consistently.*

Line 33: The verb "employing" is not appropriate for describing methods. Replace it with "applying."

*Response:* *We sincerely thank the reviewer for their attention to detail and for pointing out the inappropriate use of the verb "employing" in Line 33 of the manuscript.* *In response, we have revised the sentence in the Abstract (Line 33) by replacing "employing" with "applying" to accurately describe the application of Monte Carlo simulation and health risk assessment methods in the study.*

Line 46: Choose more precise and relevant keywords.

*Response: Thank you for your valuable feedback regarding the keywords. Based on your suggestion, we have revised the keywords to be more precise and relevant to the study. The updated keywords are:*

*Keywords: Heavy metals, Arsenic, Bread contamination, Wheat flour, Health risk assessment.*

Line 69: Avoid repeating "World Health Organization"; use the acronym "WHO" after it has been defined once.

*Response: We appreciate your valuable feedback. We have revised the manuscript to ensure that "World Health Organization" is defined at its first mention, and we have subsequently used the acronym "WHO" throughout the rest of the article to avoid redundancy. Thank you for your guidance on improving the clarity and conciseness of our writing.*

Line 104: What is the per capita bread consumption in Mashhad? What types of bread are commonly produced in the city? The introduction states that "contamination with heavy metals occurs during the preparation and processing of bread."

*Response: We sincerely thank the reviewer for their valuable feedback, which has guided us in enhancing the clarity and completeness of the manuscript. The comment regarding Line 104 requests specific details about the per capita bread consumption in Mashhad, the types of bread commonly produced in the city, and an explanation of bread production processes to support the statement about heavy metal contamination during preparation and processing. We have addressed these points by revising the Introduction section. The response addresses these by revising the Introduction to include:*

1. *Per Capita Bread Consumption: Mashhad aligns with Iran's average of 420 g/day per person, higher than the global 330–410 g/day (FAO, 2020; Foodex Iran, 2024).*
2. *Bread Types: Common breads in Mashhad include Sangak, Barbari, Taftoon, and Lavash, produced in traditional and semi-industrial bakeries* *using wheat flour (Haghayegh, 2016).*

Line 107: The authors discuss industrial growth around Mashhad and its potential impact on agricultural contamination.

*Response: Thank you for your valuable comment. Based on your valuable comment, introduction was revised as follow:*

*Introduction*

*Food contamination with arsenic and heavy metals due to factors such as bioaccumulation, persistence in the environment, longevity, and toxicological characteristics significantly impacts human health and can lead to various diseases and complications. This issue is one of the foremost concerns regarding food security and safety in human societies [1, 2] . In this context, organizations such as the European Commission (EC), the World Health Organization (WHO), Food and Agriculture Organization (FAO), and various regulatory bodies in different countries strictly regulate the permissible concentration or maximum allowable limits of toxic heavy metals in food products [3].*

*Risk assessment, as developed by the U.S. Environmental Protection Agency (EPA), provides a framework for estimating potential health risks from environmental pollutants. It involves identifying and quantifying the likelihood of adverse health effects from specific chemical exposures, considering all relevant exposure pathways [4]. According to WHO and the International Programme on Chemical Safety, risk assessment estimates the probability of adverse health effects from current and future exposure to environmental chemical pollutants in organisms, systems, or populations[5].*

*Heavy metals such as arsenic occur naturally but can also originate from human activities like industrial processes, agriculture, mining, and traffic emissions [6, 7]. The primary exposure pathway for humans is through consumption of contaminated foods [8, 9]. Bread, a staple worldwide rich in energy, fiber, and nutrients [10-13], is particularly vulnerable to contamination from soil, irrigation, fertilizers, milling, and processing additives (e.g., water, salt) [14, 15].*

*Iran ranks second globally in per capita bread consumption, with an intake approximately double that of European nations [16]. Global statistics indicate an average daily bread consumption of 330–410 grams per person, whereas in Iran, this figure is elevated, averaging 420 grams per person [17]. Common bread types in Mashhad include traditional Iranian varieties such as Sangak (a whole wheat sourdough baked on pebble stones), Barbari (a thick, yeast-leavened flatbread), Taftoon (a thin, soft flatbread), and Lavash (a thin, unleavened flatbread), which are produced in both traditional and semi-industrial bakeries with wheat flour [18].*

*Sources of contamination in flour and bread are diverse, encompassing agricultural, industrial, and post-harvest factors. During wheat cultivation, heavy metals may enter crops through contaminated soil, often influenced by natural geochemical processes or anthropogenic activities such as mining, industrial emissions, and urban runoff [19]. Irrigation water, particularly groundwater, is a major source of arsenic in wheat, especially in regions like Iran where contaminated aquifers are used for agriculture [20]. The application of phosphate fertilizers and pesticides further introduces cadmium and lead into soils, which are taken up by wheat plants [21]. Beyond raw materials, individual ingredients used in bread production can contribute to heavy metal levels. Flour, as the primary component, is the dominant contamination pathway due to its direct link to wheat cultivation, but other ingredients such as salt, yeast, and food additives may also play a role. Salt, often sourced from natural deposits or seawater, can contain trace amounts of heavy metals like lead and cadmium, depending on its purity and origin [22]. Yeast, while typically low in heavy metals, may introduce contaminants if produced in facilities with contaminated water or substrates. Food additives, such as improvers or preservatives, can contain heavy metals as impurities, particularly if derived from mineral-based sources [23]. Post-harvest processing, including milling and baking, can further contribute through contact with metal-containing equipment or contaminated water used in dough preparation [24].*

*Research providing detailed insights into heavy metal levels, daily intake, and associated risks in bread is crucial for safeguarding public health and improving food safety policies. While previous studies in Iran have extensively examined heavy metals in wheat and related crops [25-27], research specifically focused on bread remains limited, particularly in metropolis as Mashhad. This study addresses a significant gap by providing the first health risk assessment of heavy metal exposure from bread consumption in Mashhad, a major city in Northeast Iran. Despite Iran's high per capita bread consumption and the vulnerabilities of local bakeries, no such investigation has been conducted in this region to date. By evaluating heavy metal concentrations, intake levels, and risk indices, this research extends beyond previous studies focused solely on wheat, offering new insights to improve food safety and public health in a uniquely challenged urban environment.*

However, they should clarify what percentage of the wheat or flour used in the city is locally sourced.

*We sincerely thank the reviewer for their insightful comment requesting clarification on the percentage of wheat or flour used in Mashhad that is locally sourced. We acknowledge the importance of understanding the origin of wheat and flour to contextualize the sources of heavy metal contamination in bread and flour. However, there are no accurate statistics or clear patterns available regarding the proportion of locally sourced versus imported wheat or flour used in Mashhad.*

Line 111: Were samples taken from only one type of flour?

*Response: P5-Line 123-136:*

*Sampling was conducted based on the scientific sampling method with the aim of covering all areas in Mashhad city. Five locations (North, South, East, West, and Central) with 90 bakeries were selected for bread sampling based on their location and use. The sample size was calculated using a factorial approach. Considering the inclusion of 90 bakeries and three types of samples from each bakery (flour, dough, and bread), the total sample size for this research is 270 samples. Samples were collected from traditional and industrial bakeries in various regions of Mashhad, Iran, between June and December 2020. All bread samples were transported under dark and cool conditions and stored at a temperature of 4 ℃ until laboratory analysis. During that time, official analytical methods (AOAC, 2012) were applying for sample preparation in the laboratory (26). To reduce moisture and ensure a consistent weight, precisely 10 g of each bread sample were subjected to drying in an oven set at 100 °C. Subsequently, all dried samples were blended, sifted through a 2-mm sieve, placed in polyethylene containers that had been previously washed twice with distilled water, and stored at room temperature for further analysis (26, 27).*

Did authors consider different types of bread in heavy metal levels? Were different types of bread also considered?

*Response: P12-Line 258-260:* *There was no* *significant difference in the levels of heavy metals in different types of bread (Lavash, Barbari, Sangak) in Mashhad (Table S2). Significant differences in heavy metals were observed in bread in different regions of Mashhad.*

The authors should explicitly state the types of bread and the different baking processes used, including oven types.

*Response:* *The primary objective of this study was to examine the levels of contaminants and the associated risks in breads produced in Mashhad city. Investigating the sources of heavy metal contamination, such as the type of oven and baking methods, was not within the scope of this research. However, we recommend that future studies focus on identifying the sources that contribute to heavy metal contamination in bread.*

Line 168 & Line 188: All parameters in the formulas should be defined clearly, one by one.

*Response: Thank you for your valuable feedback. We have revised the manuscript to include clear definitions for all parameters in Equations (1), (2), (3), and (4). Each parameter is now defined immediately after its first appearance, ensuring clarity for the readers.*

Table 1: Ensure that this table is properly referenced in the text.

*Response: We appreciate your directive to reference Table 1. We added a reference in the "Human Health Risk Assessment" subsection of "Materials and Methods": "Variables used in the risk assessment equations, including ingestion rates, exposure durations, body weights, reference doses (RfD), and oral slope factors (OSF), are detailed in Table 1."*

Line 224: It is useful to recognize heavy metals whose levels exceed the average. However, this should be clearly stated and discussed in relation to the findings presented in the table.

*Response:* *P14-16-Line 311-362:* *We sincerely thank the reviewer for their insightful comment, which has helped us improve the clarity and depth of our manuscript. In response, we have revised the manuscript to explicitly identify and discuss heavy metals whose concentrations exceed the overall mean across flour, dough, and bread samples, with direct reference to the findings presented in Table 2.* *Based on your valuable comment, discussion was revised as follow:*

*Among the 12 heavy metals analyzed, concentrations of Fe, Al, As, and Cr exceeded standard limits. In contrast, levels of Cu, Ni, Zn, Cd, Co, and V remained within permissible thresholds. Pb and Hg were below the detection limits of the analytical equipment used.*

*The elevated average concentrations of Fe and Cr beyond standard thresholds may result from soil contamination due to geological sources, overuse of chemical fertilizers on cereal crops, iron fortification of wheat flour, deterioration of processing equipment, and baking processes [50-52]. Naturally, aluminum occurs in environments through processes such as soil erosion, mineral weathering, and volcanic activity. Additionally, anthropogenic activities, including pesticide application, mining, and metallurgical industries, contribute to its presence. Contaminated water, flour, salt, yeast, food additives used in bread, aluminum utensils, and combustion from flame torches can also elevate aluminum levels in bread products [53, 54]. Arsenic contamination in bread can originate from pesticide use, organic fertilizers, and irrigation water contaminated with industrial wastewater employed in wheat cultivation. Moreover, metals such as zinc and iron often contain arsenic impurities. Consequently, the use of low-purity alloys in manufacturing equipment and metal tools in contact with dough and bread may serve as sources of arsenic contamination. High-temperature processes involved in bread production facilitate the migration of ions from metal surfaces to the food, while equipment corrosion further increases metal leaching, contributing to contamination [55, 56].*

*A study conducted in Tehran in 2014 reported that Cu, Pb, and Cr concentrations in bread samples were within standard limits. However, iron levels often exceeded WHO guidelines [57], aligning with the current study's findings. Notably, this study also observed slightly higher Pb levels than permissible standards, contrasting with the present results. Investigations in Golestan Province in 2019 [58] and Hamadan City in 2017 [15] revealed that only Cd levels exceeded WHO standards, while other metal concentrations remained within acceptable limits.*

*Previous research by Khaniki et al. (2004) in Tehran indicated that Cd and Pb levels in bread samples exceeded standard thresholds, whereas Ni concentrations were within permissible limits [59]. Similarly, Feizi et al. (2016) in Chaharmahal and Bakhtiari reported that As, Fe, Cu, Ni, and Cd levels were within safe limits, but Pb concentrations exceeded permissible thresholds [10]. Haji Mohammad et al. (2013) in Yazd found Pb and Cd levels below maximum allowable limits [60]. Ghoreishy et al. (2018) in Isfahan observed that while Cd levels remained within standards, some samples showed Pb levels above permissible limits [61].*

*Based on multiple studies across Iran from 2014 to 2019, including cities such as Tehran, Golestan, Hamadan, Yazd, Isfahan, and Kermanshah, most reported Pb and Cd concentrations exceeding safety thresholds, which contrasts with the current study’s findings. Variations in heavy metal levels across regions can be attributed to factors such as differences in wheat cultivation practices, processing methods, storage conditions, equipment quality, baking techniques, additive use, and water sources.*

*Comparative studies from other countries also provide valuable context. For instance, Christopher (2007) in Nigeria analyzed heavy metals in wheat flour and found that manganese, lead, arsenic, and mercury were not detected in all samples, with overall concentrations below acceptable limits [62]. A 2013 study in Ghana evaluated metals like Co, Cd, Fe, Mn, Cu, Zn, Pb, Ni, Cr, As, and Hg, observing significant differences in Fe, Zn, Mn, Cd, and Pb levels, while Co, Ni, Cu, Cr, As, and Hg were below detection limits [52]. The European Food Safety Authority (2012) reported that Cd levels in bread remained below regulatory standards[63] . In Poland, Filon et al. (2013) found that Pb and Cd intake levels, although substantial, stayed within WHO and FAO safety standards [64]. Similarly, a 2014 Nigerian study by Oyekunle et al. measured Co, Cu, Mn, Pb, and Zn, noting that Co, Mn, and Pb exceeded WHO and FAO guidelines, while Cu and Zn did not [65]. In China, Lei (2015) assessed heavy metals in wheat flour, finding most concentrations below Chinese safety thresholds, though approximately 15% of samples exceeded standards [66]. Magomya (2013) in Nigeria reported that all heavy metals measured in bread samples were within permissible limits, except for Fe in two samples [67]. Studies from Ethiopia [68] and Spain [69] similarly reported metal levels within regulatory limits .*

Line 254: What types of ovens were considered, and how do they influence heavy metal levels? Were formulation effects also considered? It need to be clearly explained.

*Response: We appreciate the reviewer’s comment, which has prompted us to clarify the scope of our study. A detailed investigation of oven types or formulation effects was not among the goals of our study, as the main goal was to investigate the level of heavy metal contamination in bread and assess the risk arising from it.*

Figure 2 & Table 2: Data should not be duplicated. The authors should either remove Table 2 or Figure 2 to avoid redundancy.

*Response: We thank the reviewer for highlighting the issue of duplicated data between Figure 2 and Table 2. To address this concern, Table 2 was moved to the Supplementary Materials as Table S2, and all references to Table 2 in the manuscript have been updated to refer to Table S2. Figure 2 has been retained in the main text to provide a clear visual representation of the heavy metal concentrations, while Table S2 offers detailed numerical data for readers seeking additional specificity.*

Line 278: Replace "(See table S2)" with "(Table S2)".

*Response: Thank you for your suggestion. We have revised the text to replace "(See table S2)" with "(Table S2)" to enhance clarity.*

Lines 334–338: Some sentences are unclear and need to be rewritten for better readability.

*Response: We sincerely thank the reviewer for their comment, which has guided us to improve the readability of our manuscript. To address this concern, we have revised unclear sentences in the Introduction, Materials and Methods, Results, and Discussion sections by simplifying complex phrasing, splitting long sentences, and clarifying ambiguous terms. These changes enhance the manuscript’s clarity and accessibility.*

Line 388: Was the effect of water quality on heavy metal levels in bread considered? If not, why was this factor excluded?

*Response:* *We appreciate the reviewer’s comment, which has prompted us to clarify the scope of our study. Investigating the source of heavy metal contamination, including the effect of water quality, was not one of the objectives of this study. The main objective was to investigate the amount of heavy metals in breads in Mashhad and assess the resulting risk. According to our results, the highest level of heavy metal contamination was identified in flour (83 percent), and only a small amount of contamination (17 percent) was related to additives (dough and water) and oven type (Figure S1).* *However, we recommend that future studies focus on identifying the sources that contribute to heavy metal contamination in bread.*

Line 425: Does wheat variety affect heavy metal accumulation? If so, this should be discussed.

*Response:* *We thank the reviewer for their insightful comment, which has prompted us to clarify the scope of our study. Investigating the effect of wheat variety on heavy metal accumulation was not one of the goals of our study. The primary objective was to quantify the levels of heavy metals in breads in Mashhad and assess the resulting health risks.*

Lines 427–453: The references provided seem to be a general report rather than a structured conclusion. The authors should clarify whether they are using these references to support their findings or simply listing them without purpose.

*Response:* *We thank the reviewer for their valuable comment, which has prompted us to enhance the clarity and structure of the Human Health Risk Assessment section. To address the reviewer’s concern, we have revised the section to ensure that each reference is clearly linked to specific findings or used for contextual comparison, avoiding a general report-like presentation. The revised section presents our results first, followed by a focused discussion that integrates references to support our findings on non-carcinogenic and carcinogenic risks driven by arsenic (As) or to highlight regional and international comparisons (e.g., Ghanati et al., 2019; Huang et al., 2008; Noori et al., 2020). The discussion concludes with a clear emphasis on the need for flour quality monitoring, supported by our finding that flour contributes 83% of heavy metal contamination (Figure S1).*

***We sincerely appreciate your feedback, which has significantly enhanced the quality of our manuscript. We are confident that these revisions thoroughly address your concerns and present our results effectively. We eagerly await your approval and the opportunity to contribute to the journal's esteemed readership.***
