# Supplementary material for: Human health risk assessment of arsenic and potentially toxic elements exposure in bread and wheat flour in Northeast Iran
Source: PLoS One. 2025 Jul 23;20(7):e0327652. doi: 10.1371/journal.pone.0327652 (PMC12286368; doi:10.1371/journal.pone.0327652)
Supplement: Attachment — Submitted filename: Reviewer comments-23-11-2024.docx [file pone.0327652.s007.docx]

PONE-D-24-49990

The manuscript presents **“Human Health Risk Assessment of Arsenic and Potentially Toxic Elements Exposure in Bread and Wheat Flour in Northeast Iran,”** which is noteworthy. The subject addressed is within the scope of the journal but is based on limited and incomplete regional studies. The use of the EPA 3050B method, which is designed only for the acid digestion of sediments, sludges, and soils, is not appropriate for bread and wheat flour. Therefore, the accuracy of the data is fundamentally questionable.

Introduction- What is the novelty of the study? I could not find any significance of this work

Methodology—Sample digestion Method was not appropriate. The selection of an appropriate method for sample digestion is a critical preliminary step to ensure the acquisition of authentic and reliable results. This step is especially important as it directly influences the accuracy, precision, and reproducibility of the subsequent analytical processes.

QA/QC- This section is insufficiently reported. No specified the SRM/CRM number/name. The major drawback of this section is lack of QA/QC data, like, percentage recovery and use of SRM/CRM. The authors must provide the QA/QC data in a Table either in the manuscript or in the supplementary material.

**Presentation of Results**: This section was extremely poor. The standard permissible limits of heavy metals were not clearly mentioned. Statistical analysis and source identification, both of which are crucial for this type of popular research, were completely ignored in this manuscript.

Overall, the data authenticity, interpretation, QA/QC, sample location description, source identification, and referencing are not satisfactory for publication in the journal PLOS ONE.
